# Plasma LEAP-2 Following a Low-Calorie Diet with or without Interval Exercise in Women with Obesity

**DOI:** 10.3390/nu15030655

**Published:** 2023-01-28

**Authors:** Tristan J. Ragland, Steven K. Malin

**Affiliations:** 1 Department of Kinesiology & Health, New Brunswick, NJ 08091, USA; 2 Department of Kinesiology, University of Virginia, Charlottesville, VA 22903, USA; 3 Division of Endocrinology, Metabolism & Nutrition, Department of Medicine, Rutgers University, New Brunswick, NJ 08091, USA; 4 New Jersey Institute for Food, Nutrition and Health, Rutgers University, New Brunswick, NJ 08091, USA; 5 Institute of Translational Medicine and Science, Rutgers University, New Brunswick, NJ 08091, USA

**Keywords:** LEAP-2, interval training, hunger, satiety, obesity, diabetes

## Abstract

Liver-expressed antimicrobial peptide-2 (LEAP-2) is associated with caloric intake and glucose metabolism. **Purpose**: Assess if a low-calorie diet with interval exercise (LCD+INT) raises LEAP-2 more than LCD in relation to appetite and cardiometabolic health. **Methods**: Women with obesity were randomized to either 2 weeks of LCD (*n* = 13, ~1200 kcal/d) or LCD+INT (*n* = 12; 60 min/d) of INT at 3 min of 90% and 50% HRpeak, respectively. LEAP-2 and acylated ghrelin (AG) were measured at 0, 30, and 60 min, while glucose, insulin, C-peptide, and free fatty acids (FFA) were obtained up to 180 min of a 75 g OGTT. Fasting and 120 min OGTT appetite were assessed via visual analog scales. **Results**: LCD reduced the BMI (*p* < 0.001) compared with LCD+INT, but only LCD+INT increased the VO_2_ max (*p* = 0.04). Treatments reduced fasting LEAP-2 (*p* = 0.05), but only LCD increased LEAP-2 iAUC_60 min_ (*p* = 0.06) and post-prandial LEAP-2 stimulation (*p* = 0.02). Higher post-LEAP-2_60 min_ tended to relate to a lower desire to eat 120 min of sweet (r = 0.40, *p* = 0.07) and salty foods (r = 0.41, *p* = 0.06), as well as lower AG_30 min_ (r = −0.51, *p* = 0.01) and higher FFA iAUC_180 min_ (r = 0.56, *p* = 0.007) post-treatment. **Conclusion**: LCD, with or without INT, reduced fasting LEAP-2, but only LCD raised post-prandial LEAP-2. How diet and exercise impact LEAP-2 for lower chronic disease risk awaits further investigation.

## 1. Introduction

Obesity raises type 2 diabetes (T2D) and cardiovascular disease risk [1], with women having a higher prevalence of obesity than men [2]. Dysregulation of hunger signals is a key factor contributing to the increasing number of individuals living with obesity [3,4]. Despite interest in glucagon-like polypeptide (GLP-1), PYY, cholecystokinin (CCK), and acylated ghrelin (AG) as endocrinological factors promoting weight regulation, it is not clear that these factors are the main determinants of food intake. Liver-expressed antimicrobial peptide-2 (LEAP-2) was identified in 2003 as an AG receptor antagonist [5,6,7] by blocking ghrelin from binding to its growth hormone secretagogue receptor [5,6,7]. Since then, exogenous LEAP-2 infusion has been shown to act as a satiety signal by reducing food intake, as well as lowering plasma glucose levels, in humans [5]. Indeed, LEAP-2 deletion in mice enables ghrelin to stimulate feeding in rodents [5,6,8]. Together, this suggests LEAP-2 may be an important target of obesity through influences on eating behavior [9]. 

LEAP-2 is expressed from the small intestine and liver [9,10,11,12]. Plasma glucose is related to elevations in LEAP-2 as well [12], suggesting that nutrient ingestion may impact LEAP-2 regulation. Alternatively, negative energy balance and elevated fatty acids in the form of ketone bodies (e.g., beta-hydroxybutyrate) decrease LEAP-2 concentrations, thereby increasing food intake and weight gain in mice [13,14]. Although these findings imply that a dietary-induced manipulation of the energy balance may alter LEAP-2, few data are available examining the impact of exercise. Indeed, in one of the only published studies to date, Holm et al. reported that a single bout of endurance exercise decreased plasma LEAP-2 concentrations [14], but the implications towards food intake or metabolic health were not investigated. We recently demonstrated that interval exercise (INT) in conjunction with a low-calorie diet (LCD) suppressed post-prandial AG compared to an LCD alone in women with obesity [15]. This reduction in AG also paralleled favorable perceptions of hunger and fullness, suggesting that exercise during caloric restriction favors weight loss by influencing an orexigenic hormone known to stimulate food intake via hypothalamic action. However, it remains unknown if INT exercise in conjunction with LCD modulates LEAP-2 in relation to a perceived appetite or glucose metabolism. Therefore, we tested the hypothesis that LCD+INT would raise LEAP-2 in relation to improved appetite perception and glucose regulation.

## 2. Methods

Participants: Twenty-five females were randomized into LCD (*n* = 13, 45.2 ± 3.8 y, 37.4 ± 1.7 kg/m^2^, 19.1 ± 1.4 mL/kg/min) or LCD+INT (*n* = 12, 51.1 ± 4.0 y, 38.4 ± 2.4 kg/m^2^, 16.5 ± 0.8 mL/kg/min). This is a sub-study from a prior work assessing the effects of LCD, with and without INT, on AG and hunger [15], and some data are provided herein for ease in interpretation. One subject from our prior study [15] undergoing LCD+INT did not have remaining blood samples to assess LEAP-2 concentrations. Volunteers were recruited from the local community using social media and advertisements. All participants indicated they were weight stable (<2 kg) for a least 3 months prior to enrollment. Screenings consisted of a 12-lead electrocardiogram (EKG) exercise stress test, medical history, and physical examination, as well as blood chemistry analysis. Individuals were excluded if pregnant or had known cardiovascular disease; T2D; cancer; contraindications to exercise (e.g., musculoskeletal injuries); and/or taking medications (e.g., metformin, acarbose, GLP-1 agonists, etc.) known to affect glucose homeostasis. All participants gave their informed consent before they participated in the study. The study was conducted in accordance with the Declaration of Helsinki, and the protocol was approved by the University of Virginia Ethics Committee (IRB-HSR # 18316).

Cardiorespiratory Fitness: A cycle ergometer with indirect calorimetry (Carefusion, Vmax CART, Yorba Linda, CA, USA) was used to determine the cardiorespiratory fitness (VO_2_peak) and HRpeak as described before by our laboratory [16]. The workload was increased by about 25 watts every 2 min until the subject met volitional exhaustion, respiratory exchange ratio > 1.1, or cadence < 60 rpm. Heart rate (HR) and blood pressure were obtained at rest, while HR was monitored using a 12-lead EKG.

Anthropometrics: Participants were instructed to fast for approximately 4 h prior to body composition assessment. A digital scale was first used to measure body weight to the nearest 0.01 kg. Height was then measured with a stadiometer. Body fat and fat-free mass (FFM) was determined using the BodPod (BodPod, Cosmed, CA, USA). 

Metabolic Control: Participants were instructed to refrain from caffeine and alcohol, as well as strenuous exercise, 48 h prior to clinical testing. Participants were also asked to abstain from taking any medications or dietary supplements 24 h prior to reporting to the Clinical Research Unit. The last training bout was performed approximately 24 h before post-intervention metabolic testing.

Oral Glucose Tolerance Test: Participants arrived at the Clinical Research Unit after an overnight fast and underwent a 180 min 75 g oral glucose tolerance test (OGTT). Blood samples were obtained from an antecubital vein at 0, 30, and 60 of the OGTT for the determination of the LEAP-2 concentrations. Additional samples were collected at 0, 30, 60, 90, 120, and 180 min for glucose, insulin, C-peptide, and free fatty acids (FFA). The incremental area under the curve (iAUC) was calculated via the trapezoidal method. Post-prandial LEAP-2 stimulation was calculated as the percent change from fasting to the peak value after the glucose ingestion. The LEAP-2 to AG ratio was also calculated at 0 min and iAUC. 

Appetite Assessment: A 100 mm visual analog scale (VAS) was used at 0 and 120 min of the OGTT to assess the desire to eat salty, sweet, savory, and fatty foods. Subjects were instructed to indicate their perceived feeling by marking a single vertical line on the scale.

Low-Calorie Diet: The 2-week diet was based on preoperative recommendations for adults with obesity undergoing bariatric surgery, and details of the protocol have been previously reported [15,17]. In short, 3 days prior to the LCD intervention, participants were given a food log to categorize their habitual diet. For the duration of the 13-day intervention, participants were provided with meal replacement shakes for breakfast and lunch (Ensure^®^ Abbott Laboratories, Lake Bluff, IL, USA, 8 fl. oz; providing 160 kcal, 16 g protein, 2 g fat, 19 g carbohydrate), two 100 kcal snack options per day, and participants were instructed to consume a sensible meal (e.g., lean protein with vegetables or salad) consisting of less than 600 kcal for dinner each day. During the intervention, participants were asked to keep a detailed food log and were instructed on the proper means of cataloging their food. All the empty meal replacement containers, along with the 13-day food records were returned post-intervention to assess compliance. The difference between the average 3-day pre-intervention food logs and the 13-day average food intake was used to calculate the caloric deficit.

Interval Exercise and Low-Calorie Diet: Women randomized to undergo exercise performed 12 sessions of interval exercise (INT) over the 13-day intervention, with one rest day around day seven. All exercise sessions were supervised and consisted of cycling for 3 min intervals of 50% and 90% of heart rate peak (HRpeak) for a total of 60 min. After each exercise session, a mixed-meal shake (Ensure^®^Abbott Laboratories, Lake Forest, IL, USA, 8 fl. oz; providing 350 kcal, 13 g protein, 11 g fat, 50 g carbohydrate) was provided to offset energy expenditure exercise and match the energy deficit observed in LCD alone, as described before [18,19]. 

Biochemical Analysis: Plasma samples were immediately centrifuged for 10 min at 15,000× *g* at 4 °C and stored at −80 °C for later analyses. Fasting blood lipids (cholesterol, triglycerides, HDL, and LDL) were collected and analyzed by the University Medical Labs via colorimetric assays. Blood glucose was collected in lithium-heparinized vacutainers and immediately analyzed by a glucose oxidase assay (YSI Instruments 2700, Yellow Springs, OH, USA). EDTA tubes were used for all other blood samples. AG samples had aprotinin, dipeptidyl peptidase-4 (DPP-IV), and AEBSF (EMD Millipore, Billerica, MA, USA), with hydrochloric acid added immediately to acidify the aliquot. AG samples were analyzed using an ELISA as described before {15}. LEAP-2 peptide (i.e., LEAP-2_38-77_ and LEAP-2_37-76_) was collected with aprotinin and DPP-IV inhibitors and analyzed using ELISA (Phoenix Pharmaceuticals, Burlingame, CA, USA). Insulin and C-peptide were collected with aprotinin and analyzed via ELISA (Millipore, Billerica, MA, USA), respectively. FFA were collected in EDTA and analyzed via the colorimetric assay (Wako Chemicals, Richmond, VA, USA). To minimize inter-assay variability, pre- and post-samples for each participant were run in duplicate on the same plate.

Statistical Analysis: Analysis was performed using SPSS version 27 (IBM 26th Edition). Non-normally distributed variables were log transformed. Baseline statistics were analyzed with independent two-tailed *t*-tests. A two-way repeated measures ANOVA was used to determine between–within differences. Since fat mass reduction was statistically greater in LCD than LD + INT, it was used as a covariate to confirm treatment effects. Effect sizes were reported to assess the physiologic relevance as eta squared (η^2^) and interpreted as small = 0.01, medium = 0.06, and large = 0.14. Data are reported as mean ± SEM. Significance was set at *p* ≤ 0.05, and trends were accepted as ≤ 0.10 given these were secondary analysis.

## 3. Results

Participant Characteristics: LCD and LCD+INT reduced caloric intake over the intervention by design, and there was no difference between groups (−752.1 ± 230.4 vs. −422.7 ± 203.3 kcals/day; *p* < 0.001, η^2^ = 0.50; Table 1). As such, LCD and LCD+INT significantly reduced the BMI (*p* < 0.001, η^2^ = 0.68), although LCD reduced BMI more than LCD+INT (−0.8 ± 0.1 vs. −0.4 ± 0.1 kg/m^2^; *p* = 0.04, η^2^ = 0.18). This was explained by LCD inducing more fat mass loss (−1.2 ± 0.2 vs. −0.4 ± 0.2; *p* = 0.03, η^2^ = 0.19) than LCD+INT, since both treatments reduced FFM comparably (*p* = 0.005, η^2^ = 0.30). Only LCD+INT increased VO_2_peak (−0.5 ± 0.5 vs. 1.0 ± 0.5 mL/kg/min; *p* = 0.04, η^2^ = 0.17; Table 1).

Blood Lipids and Glucose Metabolism: LCD and LCD+INT reduced cholesterol (*p* < 0.001; η^2^ = 0.61), triglycerides (*p* = 0.002; η^2^ = 0.36), and LDL (*p* < 0.001; η^2^ = 0.51) with no change in HDL (Table 2). Although LCD and LCD+INT tended to reduce fasted glucose (−3.3 ± 2.2 vs. −2.3 ± 1.8; *p* = 0.06, η^2^ = 0.14), neither LCD nor LCD+INT influenced glucose iAUC_180 min_ (*p* = 0.94, η^2^ = 0.001). LCD and LCD+INT also tended to decrease fasting C-peptide levels (−0.2 ± 0.2 vs. −0.3 ± 0.1; *p* = 0.06, η^2^ = 0.14), with no effect on iAUC_180 min_ (*p* = 0.19, η^2^ = 0.07). While neither treatment influenced fasting insulin (*p* = 0.57; η^2^ = 0.01), both LCD and LCD+INT decreased insulin iAUC_180 min_ (−3783.09 ± 111.173 vs. −1998.33 ± 1154.73; *p* = 0.003; η^2^ = 0.33), with no difference between groups (*p* = 0.13, η^2^ = 0.09). LCD and LCD+INT both increased fasting FFA (0.02 ± 0.04 vs. 0.12 ± 0.04; *p* = 0.04, η^2^ = 0.17) but had no effect on iAUC_180 min_ (*p* = 0.30, η^2^ = 0.04).

**Figure 1:**
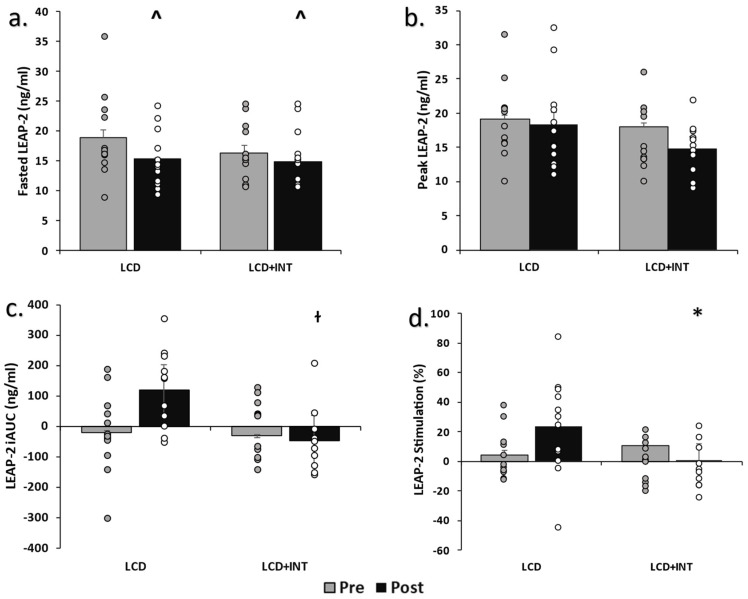
Circulating LEAP-2 before and after treatments. Note: Raw data shown as mean ± SEM, log transformed for analysis. (**a**). Fasted LEAP-2 pre- and post-intervention measurements, (**b**). LEAP-2 peak pre- and post-intervention, (**c**). LEAP-2 incremental area under the curve pre- and post-intervention, (**d**). LEAP-2 precent stimulation pre- and post-intervention, LCD: Low-calorie diet; LCD+INT: Low-calorie diet plus interval training; iAUC: incremental area under the curve; LEAP-2: Liver-expressed antimicrobial enzyme-2, Peak: highest observed value, ^ Time effect *p* = 0.05, ɫ Group × time effect, *p* = 0.06, * Group × time effect, *p* = 0.04.

Circulating LEAP-2: Both LCD and LCD+INT decreased fasting LEAP-2 (−3.4 ± 2.1 vs. −1.4 ± 1.4 ng/dL; *p* = 0.05, η^2^ = 0.16; Figure 1). However, LCD tended to increase LEAP-2 iAUC_60 min_ more than LCD+INT (*p* = 0.06, η^2^ = 0.15), and this was corroborated by LCD increasing post-prandial LEAP-2 stimulation more than LCD+INT (19.4 ± 9.1 vs. −10.3 ± 8.1%; *p* = 0.02, η^2^ = 0.20). Although neither LCD nor LCD+INT altered fasting AG (−4.6 ± 3.6 vs. 0.2 ± 4.4; *p* = 0.74, η^2^ = 0.005), LCD increased AG iAUC_60 min_ after the intervention compared to LCD+INT (162.3 ± 147.8 vs. −309.2 ± 134.7, *p* = 0.03, η^2^ = 0.19) as reported before [15]. Treatments had no effect on the fasting LEAP-2 to AG ratio (LCD: Pre: 2.1 ± 0.9, Post: 1.6 ± 0.6 vs. LCD+INT: Pre: 0.8 ± 0.1, Post: 1.2 ± 0.6; *p* = 0.58, η^2^ = 0.01) or iAUC (*p* = 0.46, η^2^ = 0.02; Figure 1) levels. 

Visual Analog Scale: LCD or LCD+INT tended to reduce fasted desire for sweets (17.0 ± 11.6 vs. 7.3 ± 6.9; *p* = 0.09, η^2^ = 0.12) and had no effect after 120 min (*p* = 0.68, η^2^ = 0.007). Neither LCD nor LCD+INT influenced fasted or 120 min desire for salty, savory, or fatty foods (Table 3). 

**Figure 2:**
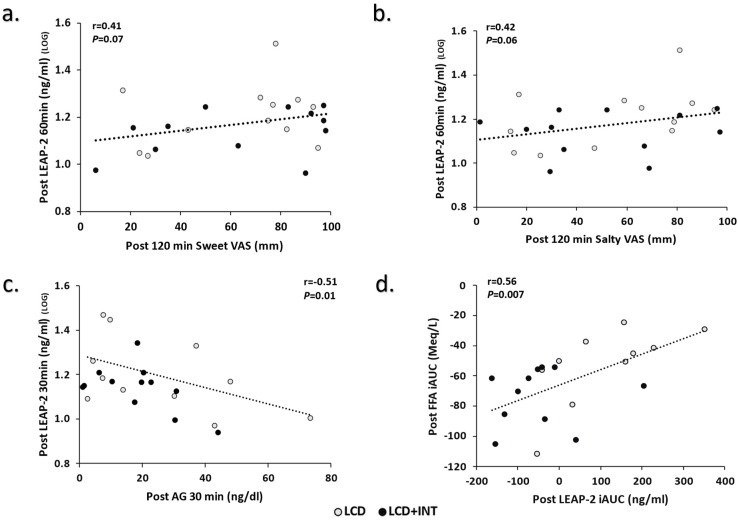
Correlations of LEAP-2 to Appetite, Acyl-Ghrelin, and Free Fatty Acids. Note: LOG indicates variables transformed for analysis, (**a**). Relationship between LEAP-2 and 120 min Sweet VAS post-intervention, (**b**). Relationship between LEAP-2 and 120 min Salty VAS post-intervention, (**c**). Relationship between 30 min LEAP-2 and 30 min AG post-intervention, (**d**). Relationship between LEAP-2 iAUC to FFA iAUC post-intervention, LCD: Low-calorie diet; LCD+INT: Low-calorie diet plus interval training; Gray circles indicate LCD; Black circles indicate LCD+INT; LEAP-2: Liver-expressed antimicrobial enzyme-2; AG: Acyl-Ghrelin; FFA: free fatty acid; iAUC: incremental area under the curve.

Correlations: Higher circulating LEAP-2_30 min_ related to lower circulating AG_30 min_ pre- (r = −0.41, *p* = 0.04) and post-intervention (r = −0.51, *p* = 0.01). There was no relationship between pre-intervention fasted LEAP-2 and fasted sweet VAS (r = −0.07, *p* = 0.71) or fasted salty VAS (r = 0.03, *p* = 0.88). Likewise, LEAP-2_60 min_ was not associated with 120 min sweet VAS (r = 0.26, *p* = 0.26) or 120 min salty VAS (r = 0.12, *p* = 0.55), respectively. However, post-intervention higher LEAP-2_60 min_ tended to associate with higher 120 min measures of sweet (r = 0.40, *p* = 0.07) and salty (r = 0.41, *p* = 0.06; Figure 2). There was no pre-intervention relationship between LEAP iAUC_60 min_ and FFA iAUC_180 min_ (r = −0.11, *p* = 0.57). Yet, higher post-LEAP-2 iAUC_60 min_ was linked to higher post-FFA iAUC_180 min_ (r = 0.56, *p* = 0.007; Figure 2). 

## 4. Discussion

The main observation of this work is, short-term caloric restriction with or without exercise decreased fasting LEAP-2 in women with obesity. However, when examining the impact of glucose ingestion on LEAP-2, it was observed that LCD raised LEAP-2 more than LCD+INT. The observed reductions in fasting LEAP-2 and rise in glucose-stimulated LEAP-2 after LCD, but not LCD+INT, is contrary to our hypothesis that exercise would raise LEAP-2 during an LCD. We anticipated such a blunted decline and rise in LEAP-2 given the literature suggests exercise may reduce appetite, as well as improve glucose regulation. Consistent with prior literature though, our work confirms that of Holm et al. [14], who reported a decreased plasma LEAP-2 during the immediate 3-h post-exercise period in healthy young males. This is consistent with the view that caloric deficit is sufficient to reduce circulating LEAP-2 levels in the fasting state and mimics a 24 h fast in mice [14] and an overnight fast in healthy men [20]. To understand the post-prandial response better though in response to approximately 25% caloric deficit, we calculated iAUC and the percent change in LEAP-2 to account for such changes in fasting LEAP-2. The observation that exercise blunted the glucose-stimulated effect on LEAP-2 is surprising, since we show here and elsewhere larger reductions in AG following LCD+INT compared with LCD [15]. In line with this idea, we observed that higher LEAP-2 was related to lower AG before and after treatment. This indicates that LEAP-2 may change in concert with AG but is not dependent on AG. Regardless, we noted that peak LEAP-2 concentrations during the OGTT are similar with fasting LEAP-2 levels at pre-treatment, confirming prior observations that glucose ingestion had little effect on post-prandial LEAP-2 up to 60 min post-ingestion, despite differences in meal energy contents (e.g., about 250 to 600 kcal) [11,12]. The novelty here is LCD and LCD+INT appear to differentially impact glucose-stimulated-LEAP-2 stimulation. Thus, our work extends on prior work using acute stimuli to induce changes in LEAP-2 [14,20] or cross-sectional data of different health status [21] by showing short-term training during an energy deficit does not alter reduction-s in LEAP-2 during the fasting state, 24 h after the last exercise bout but does potentially influence post-prandial responses among women with obesity. 

There are potential explanations for why LEAP-2 was altered post-treatment. Reductions in body weight and/or fat have been linked to lower LEAP-2 [12]. In fact, during active weight loss LEAP-2 has been reported to decline in effort to foster the AG effect on stimulating food intake [12]. Thus, it could be reasonable that lower fat mass reduces LEAP-2 gene expression from the gut and/or liver [5]. However, despite weight loss observed in both LCD and LCD+INT, we did not detect any significant relations between weight loss per se and LEAP-2. Furthermore, we covaried for fat mass loss and treatment effects remained. Instead, reductions in LEAP-2 have been linked to elevations in fat utilization [14,20,21] and ketone bodies [14]. We did not measure ketone bodies in our study per se, but we did detect higher circulating fasting FFAs following both LCD and LCD+INT and prior work by our group reported elevated fasting fat oxidation [18]. To this end, LCD+INT raised metabolic flexibility (i.e., fasting to glucose-stimulated carbohydrate oxidation) compared with LCD [18]. Thus, it would seem reasonable to anticipate higher ketone bodies in our study impacted LEAP-2 during LCD uniquely from LCD+INT and additional work investigating how ketone production may modulate fasting LEAP-2 is warranted given FFA correlated with LEAP-2 post-intervention only. 

We also recognize that the reduced carbohydrate oxidation during the OGTT in LCD post-treatment from our prior work may hint at glycogen storage vs. oxidation being a factor regulating LEAP-2 during the post-prandial period. Indeed, during energy deficit LEAP-2 has been reported to decline in effort to raise blood glucose for the prevention of hypoglycemia via a potential influence of glucagon mediated hepatic glucose production [12]. This would be consistent with observations that higher plasma LEAP-2 has been associated with higher HbA1c in T2D [21]. In our work, fasting glucose levels tended to decrease post-treatment, as did C-peptides. This suggests hepatic insulin sensitivity may have improved and serve as one possible mechanism explaining the reductions in fasting LEAP-2 levels. However, the lack of statistical difference between groups in post-prandial insulin is interesting, since effect size calculations suggest moderate relevance. We interpret this work with caution, as insulin seemingly declined more after LCD than LCD+INT, with no difference in post-prandial glucose between groups. This would suggest potential differences in either how bodily tissues respond to insulin, insulin secretion, or clearance of insulin itself from the liver. Prior work demonstrated that in vitro LEAP2_38-47_ fragment had insulinotropic effects in a human pancreatic pseudo-islet glucose-stimulated insulin secretion assay [11]. However, the translation of such work in humans is uncertain as LEAP2_38-47_ infusion had no apparent insulin secretory changes during a graded glucose clamp in healthy, young male participants of normal weight status [11]. In support of the later mechanism, C-peptides were not different between groups post-treatment in our study, although effect size calculations suggest moderate relevance after LCD compared to LCD+INT. This suggests either reductions in insulin secretion and/or liver clearance could have related to LEAP-2. Additional work is required to test such speculation. 

LEAP-2 has gained interest as a potential pharmaceutical drug given exogenous administration suppresses hunger and decreases food intake [5,20]. While many studies investigating the effects of exercise on hunger and/or satiety have focused on AG [15,17,22] or other gut-derived hormones (e.g., GLP-1, PYY, etc.) [17,22,23,24,25], a novel aspect of our study is the investigation of exercise on circulating LEAP-2 during an LCD. A consideration of prior studies though infusing LEAP-2 often used supraphysiological concentrations, thereby limiting direct extrapolation to human intervention trials. Nonetheless, LEAP-2 may decrease food intake by opposing ghrelin from binding to growth hormone secretagogue receptor and eliciting desires to eat [12]. We report that LEAP-2 may impact appetite specifically since it related to reduced desires to consume sweet and salty foods. This relationship suggests that during the early phase of weight loss by diet, with or without exercise, higher LEAP-2 may relate to a lower desire for energy dense foods that relate to obesity [26,27,28]. Interestingly, LEAP-2 correlated with AG with pre- and post-treatment correlations, and we observed no difference in LEAP-2 to AG ratio, highlighting LEAP-2 may work through similar mechanisms as ghrelin. Additional work is needed to fully elucidate the mechanism(s) by which LEAP-2 may influence the brain centers implicated in food choices [8]. Indeed, while human work examining LEAP-2 on brain areas awaits further studies, rodent studies of c-fos expression show that orexigenic neuropeptide Y neurons of the hypothalamus are inhibited by LEAP-2 [10,12]. In fact, in mice fed ad libitum, intracerebroventricular co-administration of LEAP-2 and neuropeptide Y reduced food intake compared with intracerebroventricular neuropeptide Y administration only [20]. In contrast, LEAP-2 overexpression has been reported to increase pro-opiomelanocortin (POMC) mRNA/protein and intracerebroventricular LEAP-2 administration increased c-fos activity in POMC neurons, thereby highlighting potential anorexigenic effects on neurons to reduce body weight [29]. This is consistent with work demonstrating through use of a fluorescent-labelled *n*-terminal LEAP-2 fragment, that LEAP-2 binds to growth hormone secretagogue receptor-1a expressing brain regions (e.g., hypothalamic nuclei, hippocampal dentate gyrus, and medulla) to inhibit food intake [30]. Together, these studies highlight that LEAP-2 may have direct action on the hypothalamic NYP and POMC neurons to regulate appetite. 

It is important to consider limitations in the current work. The energy deficit of the current investigation was over the course of 2 weeks and the results may change with a longer caloric deficit. LCD lost more body fat than LCD+INT and this could impact our outcomes. Why LCD lost slightly more weight/fat is beyond the scope of this analysis but could be due to either estimating energy expenditure from exercise and refeeding an absolute amount of calories post-exercise or changes in sedentary behavior. Either way, we covaried for fat loss, and this did not impact LEAP-2. This confirms that, despite both groups losing weight, and LCD inducing approximately 0.8 kg more fat loss than LCD+INT, there was no influence on LEAP-2. Testing was performed within 24 h from the last exercise session, and LEAP-2 kinetics during the immediate to longer post-exercise period (e.g., 3 to 72 h) is unknown. LEAP-2 were collected at 0, 30, and 60 min of the OGTT and it is possible we missed the exact effect of circulating and changes in LEAP-2 on the VAS measurement. However, insulin, ghrelin, GLP-1 and other appetite hormones often peak or nadir in this same time period [15,17] suggesting LEAP-2 may as well. Nonetheless, further studies are needed measuring LEAP-2 for extended periods (e.g., up to 180 min). We focused on women and cycling exercise. Therefore, it is not clear if men would respond the same way, nor if other modes of exercise would influence LEAP-2. Lastly, the assay used herein determined LEAP-2_38-77_ and LEAP-2_37-76_ peptide fragments in aggregate. However, the regulator steps in converting LEAP-2 to “active” molecules is critical towards moving the physiology field forward, and some prior works have specifically studied the LEAP-2_38-47_ fragment [11]. 

In conclusion, 2 weeks of a LCD, with and without interval training lowered fasting LEAP-2. However, LCD raised glucose-stimulated LEAP-2 compared with LCD+INT. This suggests diet and exercise may influence post-prandial LEAP-2, while energy deficit is an important regulator of fasting LEAP-2. In either case, lower LEAP-2 was related to reduced desires for sweet and salty foods post-intervention only. Together, these observations suggest LEAP-2 may play a role in calorically dense food choices and glucose regulation. Thus, future work is needed to understand how LEAP-2 may impact food intake and plasma glucose following lifestyle therapy to prevent/treat obesity, T2D, and CVD. 

## Figures and Tables

**Table 1:** Body composition, Fitness, and Dietary Intake.

	LCD		LCD+INT		T	G × T
N	13		12		-	-
Age (y)	46.2 ± 3.5		51.3 ± 3.8		-	-
Body Composition and Fitness	Pre	Δ	Pre	Δ		
Weight (kg)	102.2 ± 4.6	−2.5 ± 0.4	105.7 ± 6.0	−1.5 ± 0.4	<0.001	0.07
Body Mass Index (kg/m^2^)	37.5 ± 1.5	−0.8 ± 0.1	37.7 ± 2.1	−0.5 ± 0.1	<0.001	0.04
Waist Circumference (cm)	114.1 ± 3.5	0.5 ± 3.8	114.1 ± 4.3	−1.7 ± 1.3	0.46	0.19
Fat Free Mass (kg)	52.7 ± 2.0	−0.9 ± 0.3	53.7 ± 2.5	−0.7 ± 0.4	0.005	0.75
Fat Mass (kg)	49.0 ± 3.3	−1.2 ± 0.2	51.6 ± 4.1	−0.4 ± 0.3	<0.001	0.03
VO_2_peak (mL/kg/min)	18.9 ± 1.2	−0.6 ± 0.5	17.6 ± 1.1	0.7 ± 0.5	0.55	0.04
Dietary Intake						
Calories (kcal/day)	1939 ± 193.0	−529.6 ± 194.7	2287.8 ± 195.0	−707.6 ± 209.9	<0.001	0.56
Relative Calories (kcals/kg/day)	19.6 ± 2.2	−5.2 ± 1.9	22.4 ± 2.4	−6.7 ± 2.2	<0.001	0.61
Carbohydrate (g/day)	228.3 ± 22.8	−36.3 ± 24.7	266.1 ± 32.8	−62.2 ± 30.3	0.03	0.62
Relative Carbohydrates (g/kg/day)	2.3 ± 0.0	−0.3 ± 0.2	2.6 ± 0.3	−0.5 ± 0.3	0.04	0.56
Fat (g/day)	80.9 ± 8.9	−38.3 ± 8.2	96.7 ± 7.7	−42.9 ± 8.3	<0.001	0.70
Relative Fat (g/kg/day)	0.8 ± 0.1	−0.3 ± 0.1	0.9 ± 0.1	−0.4 ± 0.1	<0.001	0.91
Protein (g/day)	76.5 ± 9.5	−12.6 ± 8.3	91.4 ± 7.1	−22.2 ± 9.0	0.009	0.44
Relative Protein (g/kg/day)	0.7 ± 0.1	−0.1 ± 0.0	0.9 ± 0.1	−0.2 ± 0.1	0.01	0.48

Note: Data expressed as mean ± SEM. VO_2_max: maximal oxygen uptake. Relative calories and macronutrients divided by body mass in kilograms. Change scores (Δ) calculated Post minus Pre. T: *p*-value for time effect, G × T: *p*-value for group × time effect.

**Table 2:** Blood Lipids and Glucose Metabolism.

	LCD		LCD+INT		T	G × T
N	13		12		-	-
Blood Lipids	Pre	Δ	Pre	Δ		
Cholesterol (mg/dL)	204.2 ± 17.1	−29.4 ± 5.8	192.2 ± 11.1	−23.2 ± 6.5	<0.001	0.48
Triglycerides (mg/dL)	118.8 ± 15.7	−17.6 ± 9.8	109.5 ± 13.7	−33.9 ± 10.6	0.002	0.27
LDL (mg/dL)	137.0 ± 14.9	−23.3 ± 6.0	123.4 ± 9.9	−15.7 ± 5.1	<0.001	0.35
HDL (mg/dL)	47.4 ± 2.7	−3.3 ± 1.2	50.6 ± 4.6	−1.8 ± 3.4	0.16	0.68
Cholesterol/HDL (mg/dL)	4.4 ± 0.3	−0.3 ± 0.2	3.9 ± 0.2	−0.3 ± 0.2	0.01	0.93
LDL/HDL (mg/dL)	2.9 ± 0.3	−0.3 ± 0.2	2.5 ± 0.2	−0.2 ± 0.1	0.03	0.63
Fasted Free Fatty Acids (mM)	0.5 ± 0.1	0.0 ± 0.1	0.5 ± 0.0	0.1 ± 0.0	0.04	0.17
Free Fatty Acids iAUC_180_ (mM)	−60.5 ± 5.4	2.7 ± 6.7	−60.6 ± 4.6	−13.3 ± 7.0	0.30	0.11
Glucose Metabolism						
Fasted Glucose (mg/dL)	96.6 ± 1.9	−3.3 ± 2.2	99.3 ± 1.7	−2.3 ± 1.8	0.06	0.83
Glucose iAUC_180_ (mg/dL)	3537.1 ± 981.7	−544.1 ± 858.1	4214.6 ± 969.3	469.7 ± 527.3	0.94	0.33
Fasted Insulin (µU/mL)	19.2 ± 3.9	−3.2 ± 2.9	17.8 ± 4.1	1.5 ± 4.3	0.73	0.37
Insulin iAUC_180_ (µU/mL)	14,067.6 ± 1714.2	−3783.0 ± 111.1	14,687.3 ± 3527.5	−1998.3 ± 1154.7	0.003	0.23
Fasted C-peptide (ng/mL)	2.6 ± 0.3	−0.2 ± 0.2	2.3 ± 0.3	−0.3 ± 0.1	0.06	0.83
C-peptide iAUC_180_ (ng/mL)	1119.4 ± 95.2	−102.7 ± 82.7	1033.6 ± 90.3	10.02 ± 46.2	0.07	0.13

Note: Data expressed as mean ± SEM. LDL: low-density lipoprotein; HDL: high-density lipoprotein; iAUC: incremental area under the curve; HOMA-IR: Homeostatic model of assessment for insulin resistance; change scores (Δ) calculated Post minus Pre. T: *p*-value for time effect, G × T: *p*-value for group × time effect.

**Table 3:** Perceived Appetite.

	LCD		LCD+INT		T	G × T
N	13		12		-	-
Appetite	Pre	Δ	Pre	Δ		
Fasted Sweet (mm)	46.1 ± 7.2	17.0 ± 11.6	49.9 ± 7.3	7.4 ± 6.9	0.09	0.49
120 min Sweet (mm)	62.4 ± 5.9	4.3 ± 8.4	63.7 ± 8.3	−0.2 ± 4.6	0.68	0.64
Fasted Salty (mm)	52.0 ± 6.8	−6.1 ± 12.7	62.3 ± 7.6	2.7 ± 7.5	0.82	0.56
120 min Salty (mm)	58.1 ± 5.4	−3.1 ± 10.0	63.2 ± 7.3	−12.3 ± 8.8	0.26	0.50
Fasted Savory (mm)	45.6 ± 5.7	−6.2 ± 11.9	48.6 ± 8.9	1.2 ± 11.6	0.76	0.65
120 min Savory (mm)	44.0 ± 6.3	−5.1 ± 6.6	49.1 ± 8.6	−10.7 ± 11.8	0.24	0.67
Fasted Fatty (mm)	48.6 ± 7.3	7.3 ± 10.8	58.7 ± 7.1	7.6 ± 4.7	0.23	0.98
120 min Fatty (mm)	46.5 ± 5.7	2.4 ± 8.6	63.2 ± 6.7	−4.9 ± 5.5	0.81	0.48

Note: Raw data shown as mean ± SEM. VAS: visual analog scale in millimeters; change scores (Δ) calculated Post minus Pre. T: time effect, G × T: group × time effect.

## Data Availability

These data have not been made publicly available. However, the corresponding author (S.K.M.) can provide further information on the data upon reasonable request.
